# The effect of induction immunosuppression for kidney transplant on the latent HIV reservoir

**DOI:** 10.1172/jci.insight.162968

**Published:** 2022-11-08

**Authors:** Sarah E. Benner, Yolanda Eby, Xianming Zhu, Reinaldo E. Fernandez, Eshan U. Patel, Jessica E. Ruff, Feben Habtehyimer, Haley A. Schmidt, Charles S. Kirby, Sarah Hussain, Darin Ostrander, Niraj M. Desai, Sander Florman, Meenakshi M. Rana, Rachel Friedman-Moraco, Marcus R. Pereira, Shikha Mehta, Peter Stock, Alexander Gilbert, Michele I. Morris, Valentina Stosor, Sapna A. Mehta, Catherine B. Small, Karthik Ranganna, Carlos A.Q. Santos, Saima Aslam, Jennifer Husson, Maricar Malinis, Nahel Elias, Emily A. Blumberg, Brianna L. Doby, Allan B. Massie, Melissa L. Smith, Jonah Odim, Thomas C. Quinn, Gregory M. Laird, Robert F. Siliciano, Dorry L. Segev, Andrew D. Redd, Christine M. Durand, Aaron A.R. Tobian

**Affiliations:** 1 Department of Pathology and; 2 Department of Medicine, Division of Infectious Diseases, Johns Hopkins School of Medicine, Baltimore, Maryland, USA.; 3 Department of Epidemiology, Johns Hopkins Bloomberg School of Public Health, Baltimore, Maryland, USA.; 4 Department of Surgery, Johns Hopkins University School of Medicine, Baltimore, Maryland, USA.; 5 Recanati/Miller Transplantation Institute and; 6 Department of Medicine, Icahn School of Medicine at Mount Sinai, New York, New York, USA.; 7 Department of Medicine, Emory University, Atlanta, Georgia, USA.; 8 Department of Medicine, Columbia University Irving Medical Center, New York, New York, USA.; 9 Department of Medicine, University of Alabama Heersink School of Medicine, Birmingham, Alabama, USA.; 10 Department of Surgery, University of California, San Francisco, San Francisco, California, USA.; 11 Medstar Transplant Institute, Georgetown University School of Medicine, Washington, DC, USA.; 12 Department of Medicine, Division of Infectious Diseases, University of Miami Miller School of Medicine, Miami, Florida, USA.; 13 Departments of Medicine and Surgery, Divisions of Infectious Diseases and Organ Transplantation, Feinberg School of Medicine, Northwestern University, Chicago, Illinois, USA.; 14 Department of Surgery, New York University Grossman School of Medicine, NYU Langone Health, New York, New York, USA.; 15 Department of Medicine, Division of Infectious Diseases, Weill Cornell Medicine, New York, New York, USA.; 16 Department of Medicine, Drexel University, Philadelphia, Pennsylvania, USA.; 17 Divison of Infectious Diseases, Rush University Medical Center, Chicago, Illinois, USA.; 18 Department of Medicine, University of California, San Diego, San Diego, California, USA.; 19 Institute of Human Virology, University of Maryland School of Medicine, Baltimore, Maryland, USA.; 20 Department of Internal Medicine, Yale University School of Medicine, New Haven, Connecticut, USA.; 21 Department of Surgery and Transplant Center, Massachusetts General Hospital and Harvard Medical School, Boston, Massachusetts, USA.; 22 Department of Medicine, Perelman School of Medicine at the University of Pennsylvania, Philadelphia, Pennsylvania.; 23 Positive Rhetoric LLC, Bowling Green, Kentucky, USA.; 24 Department of Public Health Sciences, College of Health, Education, and Social Transformation, New Mexico State University, Las Cruces, New Mexico, USA.; 25 Department of Biochemistry and Molecular Genetics, University of Louisville, Louisville, Kentucky, USA.; 26 Division of Intramural Research, National Institute of Allergy and Infectious Diseases, NIH, Bethesda, Maryland, USA.; 27 AccelevirDx, Baltimore, Maryland, USA.

**Keywords:** AIDS/HIV, Organ transplantation, T cells

## Abstract

The HIV latent viral reservoir (LVR) remains a major challenge in the effort to find a cure for HIV. There is interest in lymphocyte-depleting agents, used in solid organ and bone marrow transplantation to reduce the LVR. This study evaluated the LVR and T cell receptor repertoire in HIV-infected kidney transplant recipients using intact proviral DNA assay and T cell receptor sequencing in patients receiving lymphocyte-depleting or lymphocyte-nondepleting immunosuppression induction therapy. CD4^+^ T cells and intact and defective provirus frequencies decreased following lymphocyte-depleting induction therapy but rebounded to near baseline levels within 1 year after induction. In contrast, these biomarkers were relatively stable over time in the lymphocyte-nondepleting group. The lymphocyte-depleting group had early TCRβ repertoire turnover and newly detected and expanded clones compared with the lymphocyte-nondepleting group. No differences were observed in TCRβ clonality and repertoire richness between groups. These findings suggest that, even with significant decreases in the overall size of the circulating LVR, the reservoir can be reconstituted in a relatively short period of time. These results, while from a relatively unique population, suggest that curative strategies aimed at depleting the HIV LVR will need to achieve specific and durable levels of HIV-infected T cell depletion.

## Introduction

One of the major challenges in finding a cure for people living with HIV (PLWH) is the persistence of the integrated provirus in latently infected cells (1). The latent viral reservoir (LVR) is established very early in infection, replenished continuously throughout viremic infection, and persists even with suppressive antiretroviral therapy (2, 3). One curative strategy proposed for HIV is to decrease or eliminate the LVR using a 2-step “kick-and-kill” strategy. This strategy relies on using latency-reversing agents (LRAs) to activate viral gene expression in latently infected resting CD4^+^ T cells, which will allow for immunological clearance (4, 5). Multiple studies have shown that specifically targeting a single reactivation mechanism is insufficient to reactivate a significant amount of the LVR, suggesting that multiple LRAs may need to be used in combination to decrease reservoir size (6–8). However, even in the presence of combinations of LRAs, attempts at kick and kill have been largely unsuccessful, which may be due in part to insufficient “killing” of infected cells by the host’s natural immune response (9–11). In addition, even if infected cells are killed, it is possible that these cells will be reconstituted by subsequent homeostatic proliferation and/or clonal expansion.

There has been interest in the potential of lymphocyte-depleting agents, such as antithymocyte globulin (ATG), for reducing the LVR by killing infected cells. ATG was part of the therapy received by the patient from Berlin who was cured of HIV with a CCR5-delta32 allogeneic stem cell transplant (12). The contribution of ATG to LVR reduction has been considered in the IciStem cohort of HIV^+^ allogeneic stem cell transplant recipients (13, 14). However, with so few patients, the effect of ATG was unclear. ATG is routinely used as induction immunosuppression in people who undergo solid organ transplantation who are considered at increased risk for allograft rejection in early posttransplant period. Those at lower risk receive nonlymphocyte-depleting IL-2 receptor antagonists, such as basiliximab, which blocks the activation of lymphocytes (15, 16).

The HOPE in Action Multicenter Consortium is a cohort of PLWH undergoing kidney transplantation from donors with and without HIV (17) (Clinicaltrials.gov, NCT02602262 and NCT03500315). Induction immunosuppression use was varied in this cohort, providing a unique opportunity to quantify and characterize changes in the LVR in PLWH receiving a potent lymphocyte-depleting or killing agent. With the introduction of the intact proviral DNA assay (IPDA), longitudinal measurements of proviral intactness within the reservoir are able to be more easily analyzed (18). T cell clones can expand in response to antigen stimulation, including cells that harbor stably integrated HIV-1 proviruses (19, 20). Although the IPDA does not allow for exploration of clonal expansion of the HIV reservoir, TCRβ sequencing provides a rich surrogate for analyzing T cell clonality and can provide insight into how induction therapies affect the total T cell population’s diversity. As such, the objective of this study was to examine changes in the LVR and T cell receptor repertoire among patients who either received T cell–depleting induction therapies or nondepleting strategies.

## Results

### Characteristics of the study participants.

There were 88 kidney transplant recipients, with a median number of 3 follow-up visits (IQR, 3–4 visits). Approximately half of the recipients, 54% (*n* = 48), received lymphocyte-depleting immunosuppression induction therapy (Table 1). Each of the groups were clinically uniform, in that they met all eligibility requirements (i.e., must be on antiretroviral therapy [ART], must be virally suppressed). However, organ allocations were pseudorandomized. The median participant age was 54 years old (IQR = 48–63 years), 20% (*n* = 18) were female, and the majority were Black 69% (*n* = 61). At time of transplant, most participants were on integrase inhibitor–containing ART therapy (97%, *n* = 85), less than half were on nonnucleoside reverse transcriptase inhibitor–containing ART therapy (33%, *n* = 29), and few were on protease inhibitor ART therapy (7%, *n* = 6). Baseline characteristics of study participants were similar among the immunosuppression induction groups (Table 1). Following transplantation, 97% (*n* = 85) of participants were taking the immunosuppressant tacrolimus.

### Longitudinal trajectories in the latent viral reservoir and CD4^+^ T cell counts.

**Figure 1:**
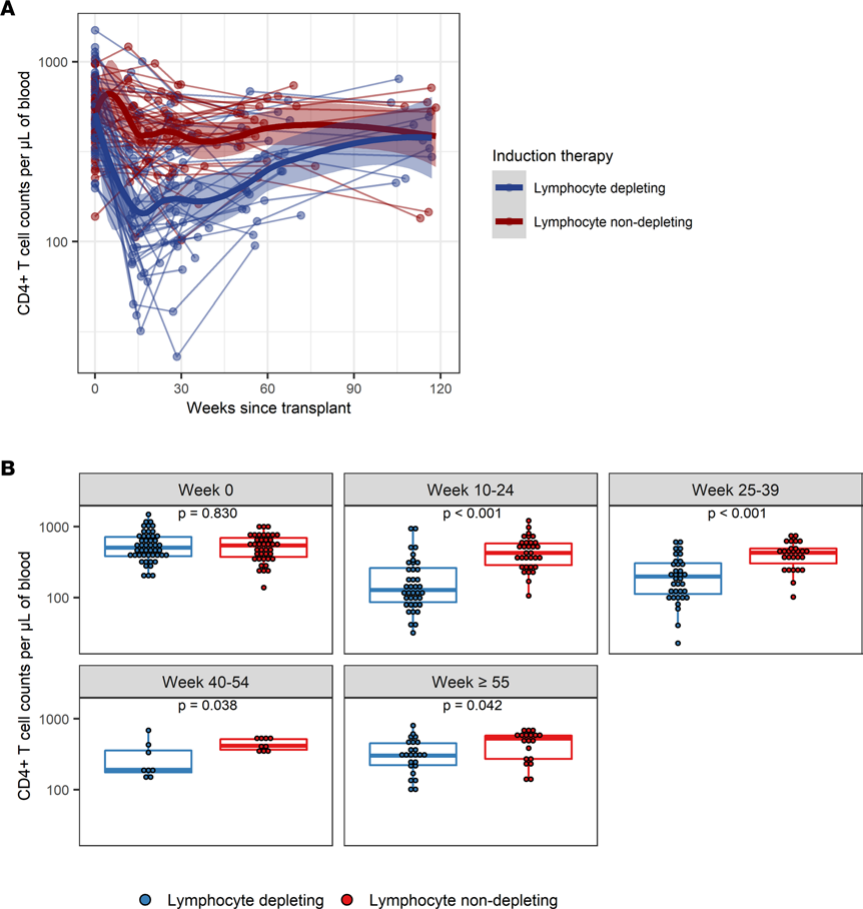
Longitudinal trajectories of CD4^+^ T cell counts per μL of blood following kidney transplant. (**A**) CD4^+^ T cell counts were measured longitudinally from time since transplant among patients who received lymphocyte-depleting or -nondepleting treatment. Each line represents an individual, and each dot represents a time point. Blue and red lines represent the locally estimated scatter plot smoothing (LOESS) curves for lymphocyte-depleting and -nondepleting groups, respectively. Gray shaded areas represent the 95% CI of the LOESS curves. (**B**) Comparisons between therapy groups were further analyzed and subdivided into time bins. Each dot represents an individual analyzed within a time bin. Box plots represent the IQR. Medians are represented by horizontal lines in the boxes. The lower and upper whiskers represent 1.5 times the IQR beyond the quartiles. *P* values were estimated by Wilcoxon’s rank-sum test.

At baseline, there was no difference in the distribution of CD4^+^ T cell amounts by lymphocyte-depleting induction therapy groups; however, CD4^+^ T cell amounts were significantly lower in lymphocyte-depleting group compared with the lymphocyte nondepleting group 10–59 weeks after transplantation (Figure 1). There was a nonlinear trajectory of CD4^+^ T cell counts over time in both the lymphocyte-depleting group and -nondepleting group. Before 25 weeks after transplant, there was a greater decline in log_10_ CD4^+^ T cell counts per week in the lymphocyte-depleting group (β = –0.0206 [95% CI, –0.0241, –0.0171]) than in the nondepleting group (β = –0.0044 [95% CI, –0.0080, –0.0007]) (difference in slope, β = –0.0163 [95% CI, –0.0213, –0.0112]) (Table 2). After 25 weeks after transplant, there was an increase in log_10_ CD4^+^ T cell counts per week in the lymphocyte-depleting group (β = 0.0062 [95% CI, 0.0045, 0.0079]) but not in the nondepleting group (β = 0.0000 [95% CI, –0.0018, 0.0018]) (difference in slope, β = 0.0062 [95% CI, 0.0037, 0.0086]).

**Figure 2:**
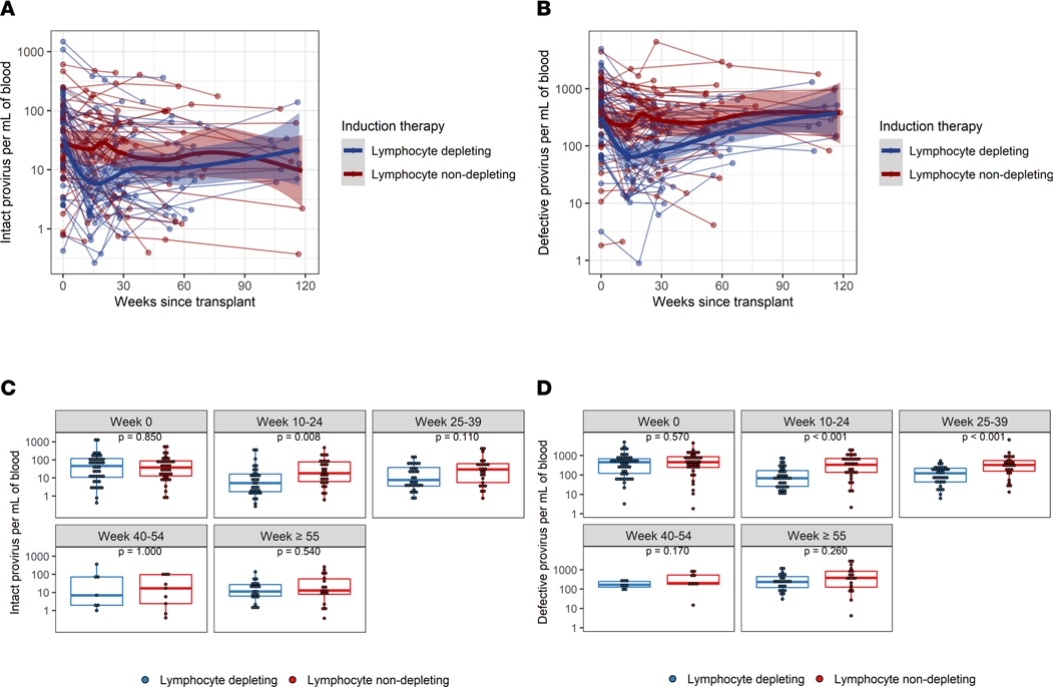
Intact and defective (3′and 5′) proviral frequencies per mL of blood following kidney transplant. (**A**) Intact and (**B**) defective proviruses were measured longitudinally from time since transplant among patients who received lymphocyte-depleting or -nondepleting treatment. Each line represents an individual, and each dot represents a time point. Blue and red lines represent the locally estimated scatter plot smoothing (LOESS) curve for lymphocyte-depleting and -nondepleting groups, respectively. Gray shaded areas represent the 95% CI of the LOESS curves. (**C** and **D**) Comparisons between therapy groups were further analyzed and subdivided into time bins. Each dot represents an individual analyzed within a time bin. Box plots represent the IQR. Medians are represented by horizontal lines in the boxes. The lower and upper whiskers represent 1.5 times the IQR beyond the quartiles. *P* values were estimated by Wilcoxon’s rank-sum test.

Baseline distribution of intact provirus and defective provirus frequencies per mL of blood were similar between lymphocyte-depleting and -nondepleting groups and became significantly different during weeks 10–24 (Figure 2). Log_10_ intact provirus frequencies declined faster over time before 25 weeks in the lymphocyte-depleting group than in the nondepleting group, and the difference of slope was –0.0153 (95% CI, –0.0257, –0.0049) (Table 2). There was a significant weekly increase of log_10_ intact provirus frequencies in the lymphocyte-depleting group after 25 weeks (β = 0.0070 [95% CI, 0.0031, 0.0109]), but this rebound was not observed in the nondepleting group (β = –0.0032 [95% CI, –0.0072, 0.0007]). Similar trajectories were observed for defective provirus frequencies; the lymphocyte-depleting group showed a greater decline in frequencies before 25 weeks after transplant, and then frequencies increased faster than the nondepleting group after 25 weeks (Figure 2 and Table 2). It should be noted that these changes are most likely a reflection of the drop in total CD4^+^ cells caused by lymphocyte-depleting treatments.

**Figure 3:**
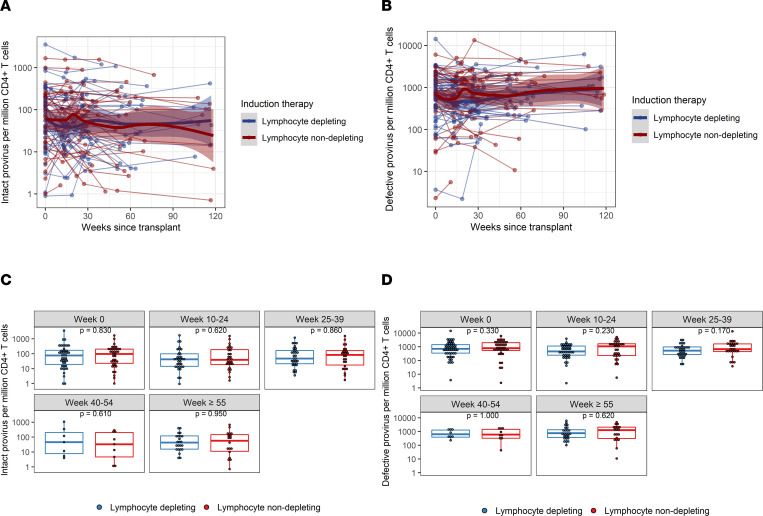
Intact and defective (3′ and 5′) provirus frequencies per million CD4^+^ T cells following kidney transplant. (**A**) Intact and (**B**) defective provirus per million CD4^+^ T cells were measured longitudinally from time since transplant among patients who received lymphocyte-depleting or -nondepleting treatment. Each line represents an individual, and each dot represents a time point. Blue and red lines represent the locally estimated scatter plot smoothing (LOESS) curve for lymphocyte-depleting and -nondepleting groups, respectively. Gray shaded areas represent the 95% CI of the LOESS curves. (**C** and **D**) Comparisons between therapy groups were further analyzed and subdivided into time bins. Each dot represents an individual analyzed within a time bin. Box plots represent the IQR. Medians are represented by horizontal lines in the boxes. The lower and upper whiskers represent 1.5 times the IQR beyond the quartiles. *P* values were estimated by Wilcoxon’s rank-sum test.

**Figure 4:**
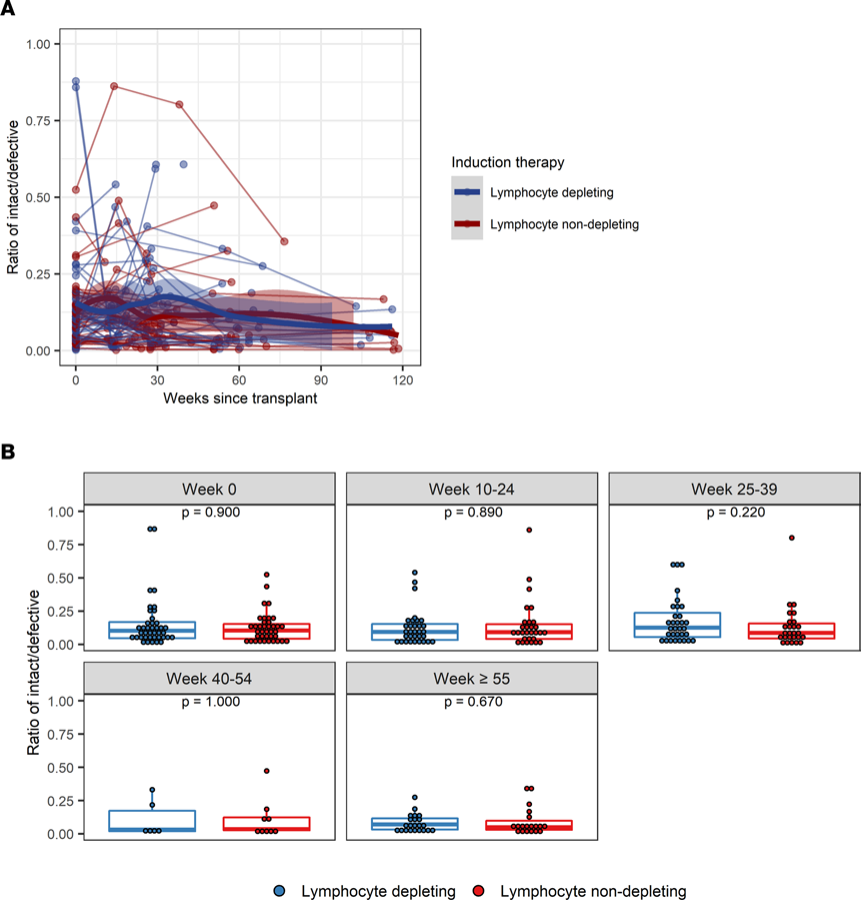
Ratio of intact/defective provirus frequencies following kidney transplant. (**A**) Intact-to-defective proviral ratios were measured longitudinally from time since transplant among patients who received lymphocyte-depleting or -nondepleting treatment. Each line represents an individual, and each dot represents a time point. Blue and red lines represent the locally estimated scatter plot smoothing (LOESS) curve for lymphocyte-depleting and -nondepleting groups, respectively. Gray shaded areas represent the 95% CI of the LOESS curves. (**B**) Ratios were further summarized by time point and compared by therapy group. Blue lines indicate participants who received lymphocyte-depleting therapy, and red lines indicate participates who received nondepleting therapy. Each dot represents an individual analyzed within a time bin. Box plots represent the IQR. Medians are represented by horizontal lines in the boxes. The lower and upper whiskers represent 1.5 times the IQR beyond the quartiles. *P* values were estimated by Wilcoxon’s rank-sum test.

The trajectory of intact provirus frequencies per million CD4^+^ T cells, defective provirus frequencies per million CD4^+^ T cells, and the ratio of intact/defective provirus was similar between the lymphocyte-depleting and lymphocyte-nondepleting groups (Figure 3 and Figure 4 and Table 2). Similar inferences were observed among patients who did not experience rejection over follow-up (Supplemental Figures 1–4; supplemental material available online with this article; https://doi.org/10.1172/jci.insight.162968DS1) within the lymphocyte-depleting therapy groups (Supplemental Figures 5 and 6), as well as between patients who received either an HIV^+^ or HIV^–^ organ (Supplemental Figures 7 and 8).

### Induction therapy effects on TCRβ repertoire.

To examine the clonal changes in the overall CD4^+^ T cell population composition after induction, a subsample of the study participants (*n* = 41) were examined using TCRβ sequencing. Of these participants, 49% (*n* = 20) received lymphocyte-depleting induction therapy. The median participant age was 53 years old (IQR, 47–59 years), 20% (*n* = 8) were female, and 71% (*n* = 29) were Black. A total of 138 samples was used, with an average of 3 visits per participant. (Supplemental Table 1).

**Figure 5:**
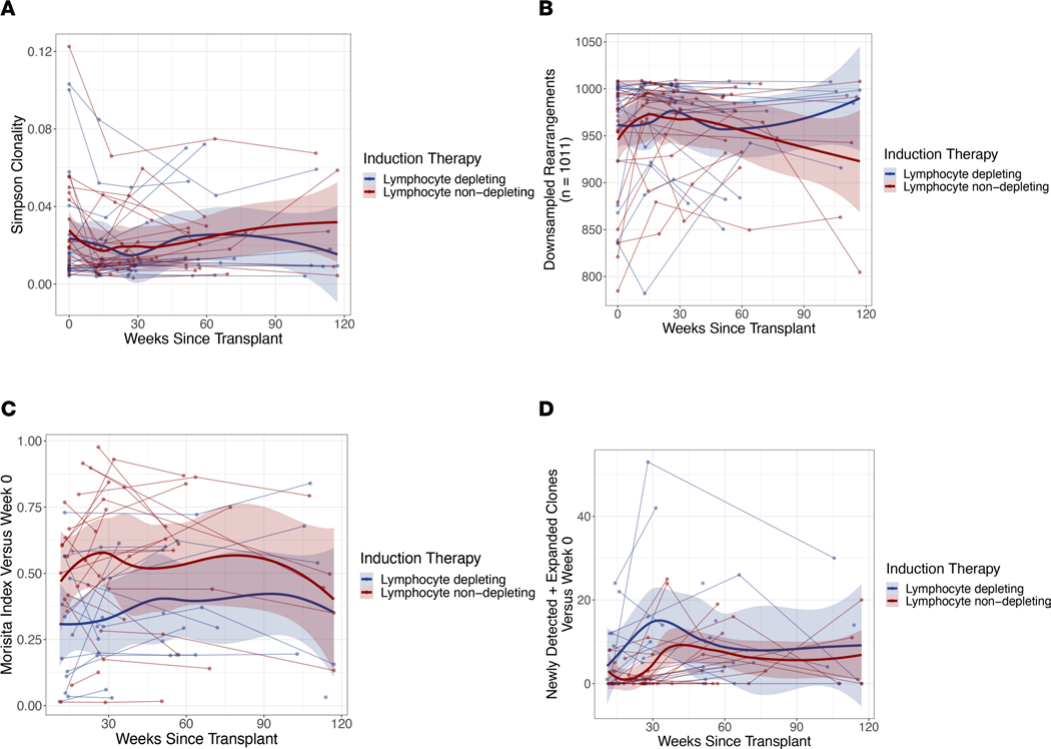
TCRβ repertoire following induction immunosuppressive therapy. (**A**) Repertoire clonality, (**B**) repertoire richness, (**C**) repertoire turnover, and (**D**) newly detected and expanded clones were measured from time since transplant among patients who received lymphocyte-depleting or -nondepleting treatments. Each line represents an individual, and each dot represents a time point. Blue and red lines represent the locally estimated scatter plot smoothing (LOESS) curve for lymphocyte-depleting and -nondepleting groups, respectively. Gray shaded areas represent the 95% CI of the LOESS curves. One patient was an outlier in **D** and, therefore, was excluded from this analysis.

No differences were observed in repertoire clonality or repertoire richness among the therapy groups at any time point following transplantation (Figure 5 and Supplemental Figure 9). Early after transplant, transplant recipients who received lymphocyte-depleting therapy had significantly higher repertoire turnover between baseline and 10–24 weeks than did patients who received lymphocyte-nondepleting therapy (*P* = 0.025); however, by 25+ weeks, repertoire turnover from baseline was not significantly different between the 2 treatment groups. Moreover, patients who received lymphocyte-depleting therapy had an increase in newly detected and expanded clones by 25–39 weeks (*P* = 0.026) compared with those who received nondepleting therapy. (Figure 5 and Supplemental Figure 9).

## Discussion

The LVR remains the main barrier to a cure for HIV. PLWH undergoing organ transplantation allow for a unique opportunity to monitor relatively large changes to the LVR over time, due to the nature of the induction therapies used to prevent organ rejection. In this study, participants treated with lymphocyte-depleting induction therapy experienced a significant initial reduction of overall intact and defective provirus that was more pronounced than any previous LRA-based study has demonstrated to our knowledge. However, the LVR was replenished to a level comparable to that of lymphocyte-nondepleting participants and baseline levels by 1 year after transplant. This initial decrease was driven primarily by the loss of CD4^+^ T cells and not selective killing of HIV-infected cells per se, as lymphocyte-depleting induction therapies have polyclonal effects on the full T cell population and are not cell specific. Moreover, even with massive declines of a log or more (>90%) in CD4^+^ T cell counts after initial lymphocyte-depleting therapy, intact versus defective proviral ratios, as well as overall CD4^+^ T cell clonality and repertoire turnover, did not change significantly, suggesting that even if a small total number of latently infected cells remains in the LVR, it is enough to reestablish the reservoir, even when a patient remains on fully suppressive ART.

Studies have shown that infected peripheral CD4^+^ T cells contribute to viral rebound (21, 22). Replenishment of CD4^+^ T cells following depletion strategies could be partially explained by clonal expansion of infected cells (23, 24). Moreover, the ratios of intact-to-defective provirus per million CD4^+^ T cells were comparable between therapy groups, and remaining defective provirus could contribute to the persistence of the LVR. Previous reports have found that some defective proviruses can be transcribed in vivo, contributing to viral protein production and immune activation (24–26). The lack of a dramatic shift in the intact-to-defective proviral ratio at later time points suggests possible homeostatic proliferation in both intact and defective proviruses. However, all patients were on immunosuppressive therapies, which could also influence intact and defective proviral rebound. Thus, more studies evaluating immunosuppressive effects on proviral homeostatic proliferation should be addressed.

PLWH have decreased CD4^+^ T cell populations, and studies have shown that those on short-term ART regimens (3 months) were unable to restore TCR diversity, compared with healthy individuals (27, 28). When evaluating TCR diversity, lymphocyte-depleting therapy correlates with early repertoire turnover, almost certainly due to the massive decrease in overall T cell populations initially, followed by clonal expansion of often newly detected TCR rearrangements to compensate for loss of T cells. Although this significant change in repertoire diversity in lymphocyte-depleting therapy–treated individuals was observed, there were no changes in the proportion of HIV-infected cells, as observed by the ratio of intact-to-defective proviral frequencies. Additionally, no differences in clonality, or repertoire richness were observed among patients treated with lymphocyte-depleting therapies. This could be partially explained by the broad targeting of lymphocyte-depleting therapy, and even with a proportional decrease in CD4^+^ T cell counts, ratios of intact to defective provirus did not change. Clonal expansions and contractions are common, and although proportions may alter overtime, clonotypes harboring inducible proviruses persist during prolonged ART (29). Although this provides a longitudinal analysis of the TCR repertoire complexity in response to induction therapies, future studies will need to link how induction therapies affect both TCR repertoire and HIV clonality.

This study has several limitations when applying these findings to the general community of PLWH. All recipients were on induction immunosuppression, which is necessary to reduce the risk of allograft rejection. However, these therapies can cause depletion of both CD4^+^ and CD8^+^ T cells. For this study, only CD4^+^ T cells were analyzed. All recipients were on various immunosuppressants following transplantation, which could affect the ability of the immune system to clear HIV-infected cells. Nevertheless, both groups appear to have equal usage of the common immunosuppressant agents. Some samples were not able to be amplified using IPDA and, thus, had to be excluded. IPDA does not provide sequencing data, and there are currently no high-throughput assays that distinguish both reservoir intactness and HIV sequence. To properly analyze HIV sequence data, an increase in cellular materials to assess the changes in the reservoir would be required; due to limitations of the amount of sample available, TCR sequencing was used as a surrogate assay. This provides a robust analysis of TCR repertoire complexity longitudinally in response to induction therapies but cannot link to HIV clonality. Of the 88 patients examined with IPDA, only 41 patients had TCRβ sequencing analysis completed. Although sequencing was used to observe repertoire diversity, TCR specificity was not measured, and this is something that should be further explored. Although the CD4^+^ T cell reservoir is the most studied LVR reservoir, there are other types of HIV reservoirs, such as myeloid reservoirs, that may have contributed to the reservoir rebound observed. Future studies will need to look at how induction therapies affect various reservoirs.

Taken together, these data demonstrate that, even in the presence of dramatic T cell depletion, which included a significant decrease in the circulating LVR, the ratio of intact-to-defective provirus did not change, and the LVR could be reconstituted in a relatively short period of time. While there are several important caveats to these findings, these data support the idea that achieving a functional cure using a kick-and-kill strategy will require both specific and durable elimination of HIV-infected T cells. Given that to date no LRA has significantly reduced the overall amount of latently infected cells in the body at the levels seen here in the lymphocyte-depleting therapy group, it is highly unlikely that this strategy alone will be successful in achieving a functional HIV cure.

## Methods

### Study population and procedures

HIV-infected kidney transplant recipients who received an organ from either an HIV-infected or uninfected donor were prospectively followed in a multicenter, nation-wide observational study and clinical trial (17, 30). Participants who had controlled HIV infection on ART, did not stop ART, had no active opportunistic infections, and had a CD4^+^ T cell count equal to or more than 200 cells per μL per federal guidelines were eligible for inclusion of kidney transplant. For HIV-infected donor eligibility, there was no criteria for minimum CD4^+^ T cell count or viral load (31). PBMCs were collected from recipients as previously described (32). Briefly, PBMCs and plasma were collected at the time of transplant (week 0) prior to immunosuppression induction therapy and at time points following transplant (approximately 13, 26, 52, and 104 weeks after transplant, if applicable). Patients must have had baseline PBMCs as well as at least 2 follow-up time points to be included in this study. Patients undergoing transplant were further evaluated by types of immunosuppressive induction therapy: either lymphocyte-depleting (i.e., ATG or alemtuzumab) or -nondepleting (i.e., basiliximab) therapy (Supplemental Table 2). Available data from baseline to 120 weeks (840 days) since transplant were included in the analysis.

For the TCRβ sequencing, patients who had at least 100 ng residual DNA remaining following the IPDA at baseline and at least 1 follow-up time point (minimum of 26 weeks after transplant) were evaluated by TCRβ sequencing.

### Laboratory testing

#### IPDA.

IPDA was performed by AccelevirDx using DNA extracted from isolated CD4^+^ T cells from cryopreserved PBMCs as previously described (18). Total CD4^+^ T cells were isolated via immunomagnetic selection (EasySep Human CD4+ T cell Enrichment Kit, Stemcell Technologies) and genomic DNA isolation (Qiagen, QIAamp DNA Mini Kit). IPDA is a duplex droplet digital PCR (ddPCR) assay that interrogates 2 informative regions in the HIV genome that are frequently deleted or hypermutation in defective proviruses (18). The first region is the packaging signal for which successful amplification produces a fluorescence in the FAM channel. The second region is the RRE amplicon within the *env* gene. Successful amplification produces fluorescence in the VIC channel. Droplets positive for FAM only are scored as 3′ defective proviruses. These include proviruses with APOBEC-3G hypermutation and/or a 3′ deletion. Droplets positive for only VIC were scored as 5′ defective proviruses. Droplets positive for both were scored as intact provirus. Double-negative droplets contained no provirus or rare proviruses with defects in both amplicon regions. To account for DNA shearing, an additional ddPCR was run, targeting a host gene with amplicons the same distance apart as the packaging and RRE amplicons. The ratio of dual-to-single fluorescence droplets for this reaction was used to calculate a DNA shearing index and correct for shearing (18).

#### TCRβ sequencing.

TCRβ sequencing was used in parallel with IPDA to measure clonal changes in T cell repertoire due to various induction therapies. Patients who had at least 100 ng residual DNA remaining following the IPDA and baseline, with at least 1 follow-up time point were used for TCRβ sequencing. TCRβ sequencing was performed with Adaptive Biotechnologies immunoSEQ assay (Survey) using genomic DNA of CD4^+^ T cells from patients to look for longitudinal changes in the diversity and clonality of the total T cell population after transplant. Clonality, richness, and repertoire turnover were determined using computational biology services from Adaptive Biotechnologies. Briefly, clonality was calculated using Simpson Clonality, where values were calculated between 0 and 1, which quantifies the range of mono- or oligoclonal dominance within the repertoire. This method weighs high-frequency clones more than other diversity metrics, making it more robust to sequencing depth. Repertoire richness was measured by computationally downsampling each sample to the lowest common template count (*n* = 1011 here) via random sampling without replacement and counting the number of unique rearrangements observed in this downsampled repertoire. After downsampling 5 times, the average was then used. Repertoire turnover was quantified using Morisita’s index. This metric accounts for how many clones were present in both repertoires and similarity of frequencies. Results ranged from 0 to 1, with 1 indicating 2 repertoires with identical clones and identical frequencies, whereas a value of 0 indicated completely unique repertoires. Finally, clonal expansion was assessed by comparing the frequencies of each unique rearrangement within a sample to the frequency of the same rearrangement in the week 0 sample from the same individual. A 2-sided binomial test was used to test the null hypothesis that the rearrangement was present at the same frequency in a later sample as in the week 0 repertoire. For this analysis, *P* values were adjusted using the Benjamini-Hochberg procedure, with a false discovery rate cutoff of 0.01. Any rearrangements passing this cutoff were identified as significantly expanded or contracted.

### Statistics

Baseline and follow-up characteristics of the study population were summarized overall and stratified by lymphocyte-depleting and -nondepleting therapy groups using descriptive statistics. In all analyses, the time metric for follow-up time was days since kidney transplant; however, results were scaled to weeks to improve interpretability, where appropriate.

Outcomes of interest included CD4^+^ T cell counts per μL of blood, as well as biomarkers of the LVR, including intact provirus, defective provirus, and ratio of intact/defective provirus frequencies. Intact provirus and defective provirus frequencies were analyzed in 2 units: frequency per mL of blood and frequency per million of CD4^+^ T cells. Provirus frequency per mL of blood was calculated using the following equation: provirus frequency per mL of blood = provirus frequency per million of CD4^+^ T cell count/10^6^.

All biomarker values, except ratio of intact/defective provirus frequencies, were log_10_-transformed to approximate normal distributions. Levels of each LVR biomarker were compared between lymphocyte-depleting and -nondepleting groups at baseline (week 0) and within categorical bins of follow-up time (10–24, 25–39, 40–54, and ≥55 weeks after transplant) using Wilcoxon’s rank-sum tests. To visualize the effect of lymphocyte-depleting therapies on the longitudinal trajectory of each LVR biomarker over time, each biomarker was plotted over time stratified by lymphocyte-depleting therapy status with locally estimated scatter plot smoothing and t-based approximation of 95% CIs. Linear slopes and corresponding 95% CIs of each LVR biomarker were estimated over time by mixed-effects linear spline models, with a knot at 25 weeks. This knot was selected based on visual inspection of the data and to allow sufficient follow-up time to examine longitudinal changes both before and after the knot. Each model included time since transplant (scaled to 7-day or 1-week periods), with a knot at 25 weeks and interaction terms with the lymphocyte-depleting group to quantify the slope of the trajectory of each outcome over time in the lymphocyte-depleting and -nondepleting groups both before and after 25 weeks, as well as to quantify the differences between these slopes. These models were adjusted for age and sex and included random intercepts and slopes assuming an unstructured covariance matrix to account for within-person correlation. Since rejection can impact the T cell response, sensitivity analyses were conducted, excluding participants who experienced kidney rejection during follow-up.

In a separate analysis, TCRβ sequencing was completed and repertoire clonality, diversity, turnover, and clonal expansion were evaluated over time in the lymphocyte-depleting and -nondepleting therapy groups both before and after 25 weeks. Samples were binned into the following discrete groups based on the time since transplant: weeks 0, 10–24, 25–39, 40–54, and ≥55. Within each bin, an unpaired Wilcox’s test was used to test for significant differences in repertoire Simpson clonality and repertoire richness between participants in the lymphocyte-depleting group and those in the nondepleting group. For all non–week 0 samples, the same procedure was used to assess differences between the lymphocyte-depleting and -nondepleting therapy groups in Morisita’s index (when compared with the corresponding week 0 sample), the number of significantly expanded clones, the number of significantly expanded clones that were also undetected at week 0, and the sum frequency of this latter group of clones. No *P* value correction procedures were applied to the results of these statistical tests.

Missing data was handled using available cases, unless specified otherwise. All analyses were performed in R 4.1 (R-Core Team) and Stata/MP (version 15.1, StataCorp).

### Study approval

The trial and current study were approved by the Johns Hopkins University School of Medicine Institutional Review Board. All transplant recipients (i.e., participants) provided written informed consent.

## Author contributions

SEB, DO, NMD, SF, MMR, RFM, MRP, SM, PS, AG, MIM, VS, SAM, CBS, KR, CAQS, SA, JH, MM, NE, EAB, BLD, ABM, MLS, CMD were responsible for data acquisition. SEB was responsible for the first draft of the manuscript. SEB, MLS, JO, TCQ, GML, RFS, DLS, ADR, CMD, AART were responsible for design of this study. SEB, XZ, EUP, RFS, CMD, ADR, AART were responsible for interpretation of the data. XZ, EUP and SH were responsible for data analysis. Administrative and logistical support was supported by YE, REF and JO. Laboratory testing procedures were provided by YE, REF, JER, FH, HAS and CSK. All authors reviewed, edited and approved the final manuscript.

## Supplementary Material

Supplemental data

## Figures and Tables

**Table 1:**
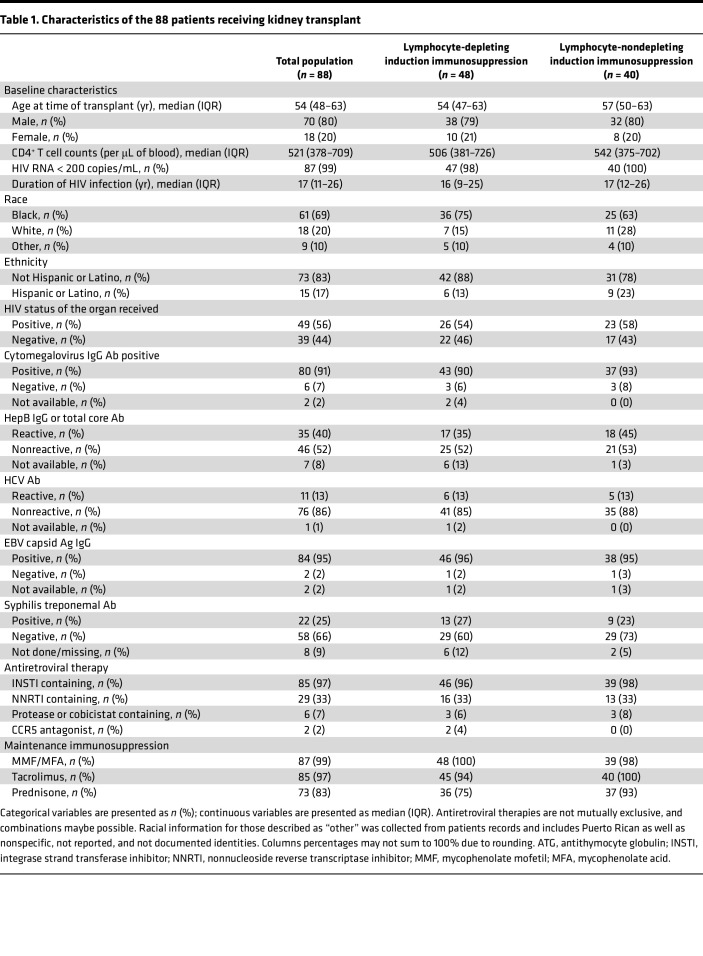
Characteristics of the 88 patients receiving kidney transplant

**Table 2:**
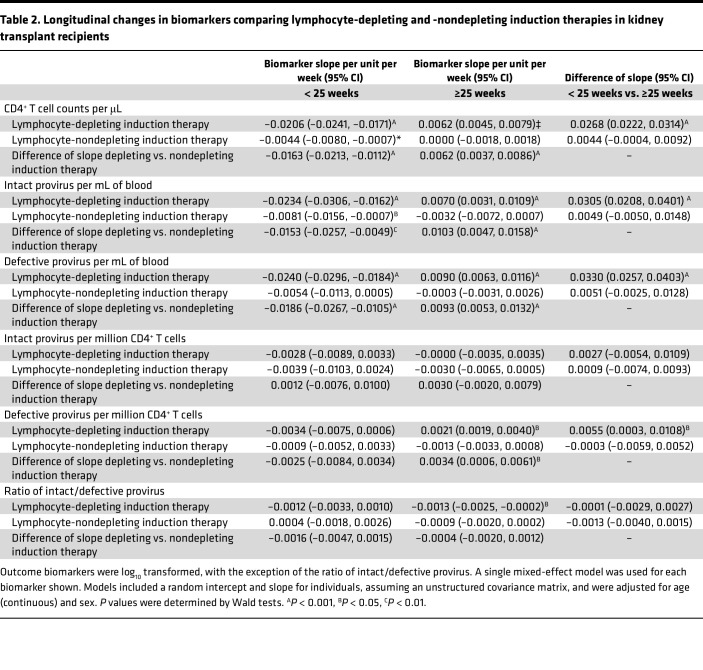
Longitudinal changes in biomarkers comparing lymphocyte-depleting and -nondepleting induction therapies in kidney transplant recipients

## Version 1 11/08/2022

Electronic publication
