# Supplementary material for: The effect of induction immunosuppression for kidney transplant on the latent HIV reservoir
Source: JCI Insight. 2022 Nov 8;7(21):e162968. doi: 10.1172/jci.insight.162968 (PMC9675561; doi:10.1172/jci.insight.162968)

SUPPLEMENTAL TABLES AND FIGURES

Supplemental Table 1. Characteristics of the 41 patients received kidney transplant in the TCRseq cohort.

	Total population	Lymphocyte depleting induction immunosuppression	Lymphocyte non-depleting induction immunosuppression
	**N=41**	**N=20**	**N=21**
**Baseline Characteristics**			
Age at Time of Transplant (years)	53 (47-59)	53 (46-58)	54 (49-60)
Male	33 (80)	16 (80)	17 (81)
CD4+ T cells count (per µL of blood)	564 (380-736)	464 (348-662)	614 (456-755)
HIV RNA < 200 copies/mL	40 (98)	19 (95)	21 (100)
Duration of HIV infection (Years)	17 (10-24)	16 (9-24)	17 (11-24)
**Race**			
Black	29 (71)	14 (70)	15 (71)
White	9 (22)	3 (15)	6 (29)
Other	3 (7)	3 (15)	0 (0)
**Ethnicity**			
Not Hispanic or Latino	33 (80)	16 (80)	17 (81)
Hispanic or Latino	8 (20)	4 (20)	4 (19)
**HIV status of the organ received**			
Positive	23 (56)	11 (55)	12 (57)
Negative	18 (44)	9 (45)	9 (43)
**Cytomegalovirus IgG Ab positive**			
Positive	39 (95)	19 (95)	20 (95)
Negative	2 (5)	1 (5)	1 (5)
Not available	0	0	0
**HepB IgG**			
Reactive	14 (34)	6 (30)	8 (38)
Nonreactive	24 (59)	11 (55)	13 (62)
Not available	3 (7)	3 (15)	0 (0)
**HCV Ab:**			
Reactive	6 (15)	2 (10)	4 (19)
Nonreactive	34 (83)	17 (85)	17 (81)
Not available	1 (2)	1 (5)	0 (0)
**EBV Capsid Ag IgG**			
Positive	39 (95)	20 (100)	19 (90)
Negative	1 (2)	0 (0)	1 (5)
Not available	1 (2)	0 (0)	1 (5)
**Syphilis Treponemal Ab**			
Positive	11 (27)	6 (30)	5 (24)
Negative	28 (68)	13 (65)	15 (71)
Not Done/ Missing	2 (4)	1 (5)	1 (5)
**Antiretroviral Therapy**			
INSTI- containing	40 (98)	20 (100)	20 (95)
NNRTI- containing	11 (27)	6 (30)	5 (24)
Protease or cobicistat containing	2 (5)	1 (5)	1 (5)
CCR5 antagonist	1 (2)	1 (5)	0 (0)
**Maintenance immunosuppression**			
MMF/ MFA	41 (100)	20 (100)	21 (100)
Tacrolimus	40 (98)	19 (95)	21 (100)
Prednisone	36 (88)	17 (85)	19 (90)

Categorical variables were presented in n (%); continuous variables were presented in median (interquartile range).
Columns percentages may not be summed to 100% due to rounding.
Abbreviations: ATG: anti-thymocyte globulin. INSTI, integrase strand transferase inhibitor; NNRTI, nonnucleoside reverse transcriptase inhibitor.

Supplemental Table 2: Types of induction therapy

	Main cohort, n = 88	TCRseq cohort, n = 41
ATG	47 (53)	19 (46)
Basiliximab	43 (49)	23 (56)
IVIG	8 (9)	4 (10)
Alemtuzumab	1 (1)	1 (2)

Abbreviations: ATG: anti-thymocyte globulin. IVIG: Intravenous immune globulin.

Induction therapies were not mutually exclusive. Eleven patients received a combination of immunosuppression induction therapy. Data were presented in n (%).

Supplemental Figure 1. Sensitivity analysis: Longitudinal trajectories of CD4+ T cells count per µL of blood following kidney transplant among patients without rejection. (A) CD4+ T cell counts were measured longitudinally from times since transplant among patients who received lymphocyte depleting or non-depleting treatment. Each line represents an individual, and each dot represents a time point. Blue and red lines represents the LOESS curve for lymphocyte depleting and non-depleting groups. Grey shaded areas represent the 95% confidence interval of the LOESS curves. (**B**) Comparisons between therapy groups were further analyzed and subdivided into time bins. Each dot represents an individual within a time bin. Boxplots represent the interquartile range (IQR). The median is represented by the horizontal line in the box. The lower and upper whiskers represent 1·5 times the IQR beyond the quartiles. P values were estimated by Wilcoxon rank sum test.

A

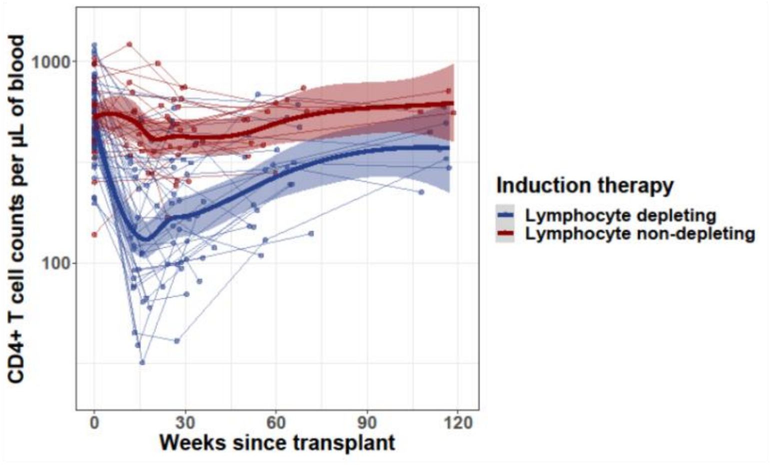

B

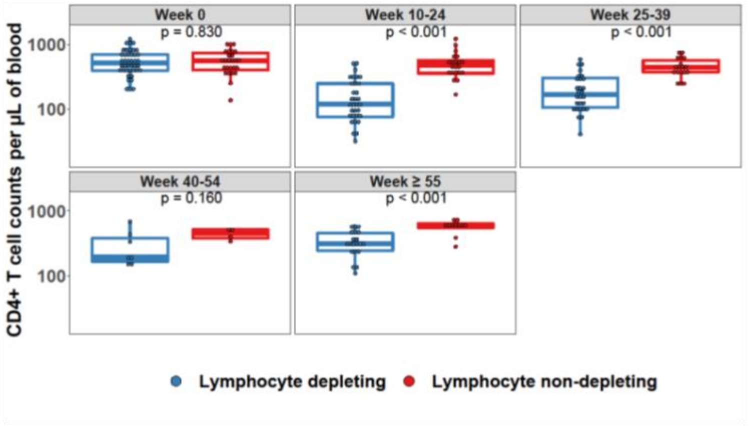

Supplemental Figure 2. Sensitivity analysis: Intact and defective (3' and 5') provirus frequencies per mL of blood following kidney transplant among patients without rejection. (A) Intact and (**B**) defective proviruses were measured longitudinally from times since transplant among patients who received lymphocyte depleting or non-depleting treatment. Each line represents an individual, and each dot represents a time point. Blue and red lines represents the LOESS curve for lymphocyte depleting and non-depleting groups. Grey shaded areas represent the 95% confidence interval of the LOESS curves. (**C,D**) Comparisons between therapy groups were further analyzed and subdivided into time bins. Each dot represents an individual within a time bin. Boxplots represent the interquartile range (IQR). The median is represented by the horizontal line in the box. The lower and upper whiskers represent 1·5 times the IQR beyond the quartiles. P values were estimated by Wilcoxon rank sum test**.**

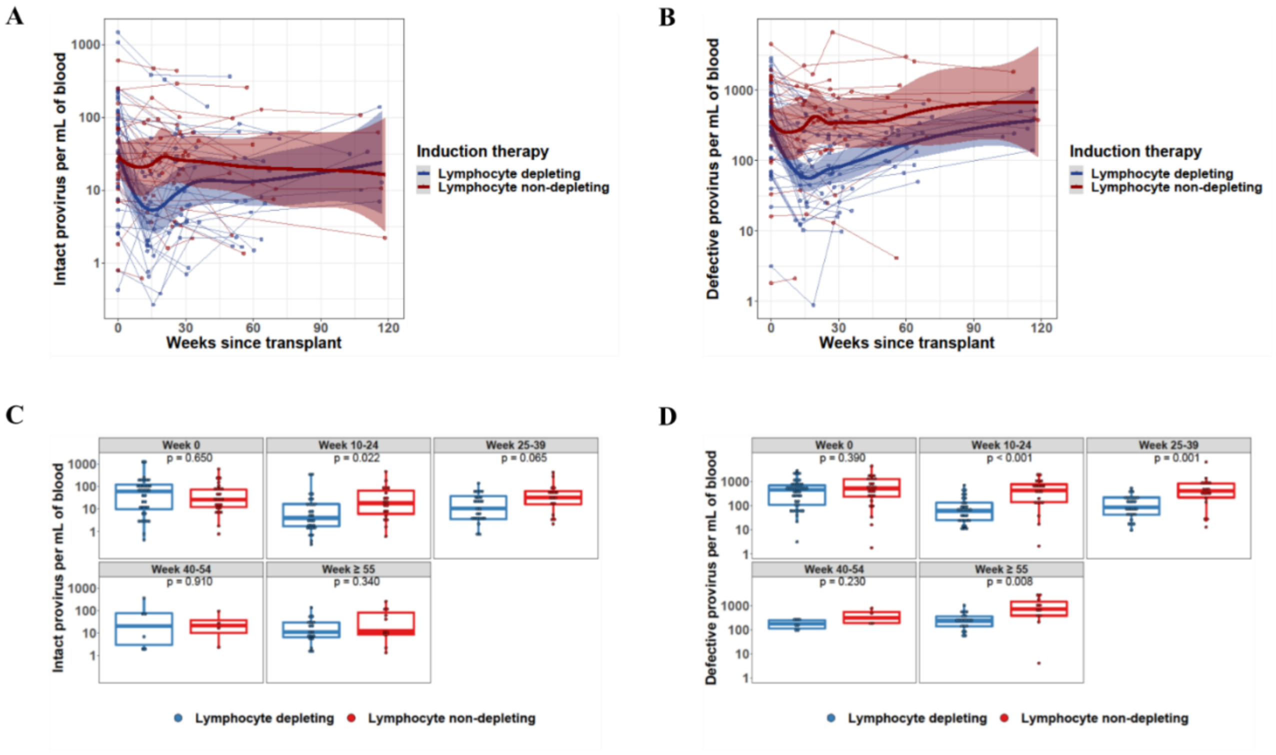

Supplemental Figure 3. Sensitivity analysis: Intact and defective (3' and 5') provirus frequencies per million of CD4$^+$ T cells following kidney transplant among patients without rejection. (A) Intact and (**B**) defective proviruses were measured longitudinally from times since transplant among patients who received lymphocyte depleting or non-depleting treatment. Each line represents an individual, and each dot represents a time point. Blue and red lines represents the LOESS curve for lymphocyte depleting and non-depleting groups. Grey shaded areas represent the 95% confidence interval of the LOESS curves. (**C,D**) Comparisons between therapy groups were further analyzed and subdivided into time bins. Each dot represents an individual within a time bin. Boxplots represent the interquartile range (IQR). The median is represented by the horizontal line in the box. The lower and upper whiskers represent 1·5 times the IQR beyond the quartiles. P values were estimated by Wilcoxon rank sum test.

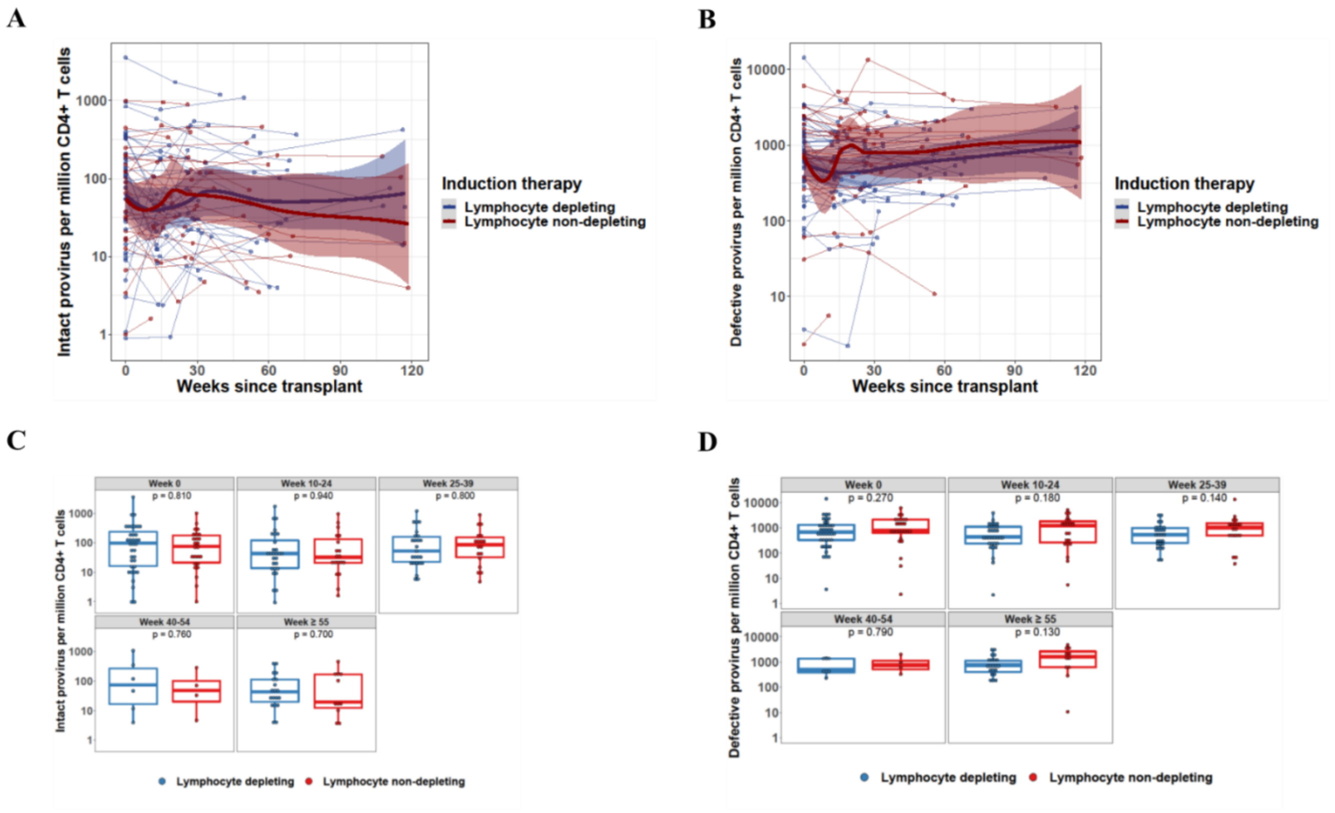

Supplemental Figure 4. Sensitivity analysis: Ratio of intact/defective provirus frequencies following kidney transplant among patients without rejection. (A) Intact to defective proviral ratios were measured longitudinally from time since transplant among patients who received lymphocyte depleting or non-depleting therapies. Each line represents an individual, and each dot represents a time point. Blue and red lines represents the LOESS curve for lymphocyte depleting compared and non-depleting groups. Grey shaded areas represent the 95% confidence interval of the LOESS curves. (**B**) Ratios were further summarized by time point and compared by therapy group. Blue lines indicate participants who received lymphocyte depleting therapy and red lines indicate participates who received non-depleting therapy. Each dot represents an individual within a time bin. Boxplots represent the interquartile range (IQR). The median is represented by the horizontal line in the box. The lower and upper whiskers represent 1·5 times the IQR beyond the quartiles. P values were estimated by Wilcoxon rank sum test.

A

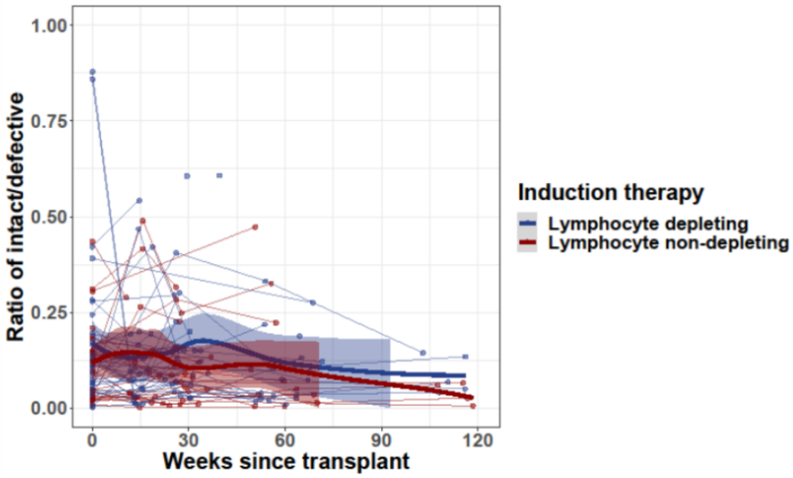

B

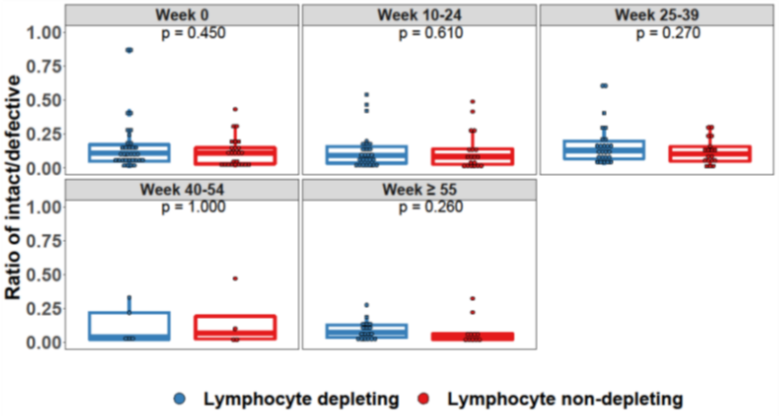

Supplemental Figure 5. Longitudinal trajectories of CD4+ T cells count per µL of blood following kidney transplant within therapy groups. Comparisons within therapy groups were further analyzed and subdivided into time bins. Each dot represents an individual within a time bin. Boxplots represent the interquartile range (IQR). The median is represented by the horizontal line in the box. The lower and upper whiskers represent 1·5 times the IQR beyond the quartiles. P values were estimated by Wilcoxon rank sum test.

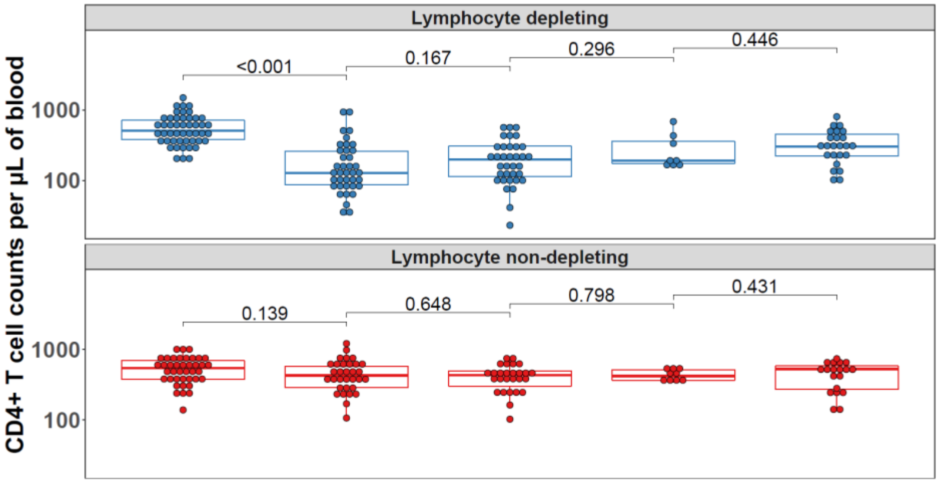

Supplemental Figure 6. Ratio of intact/defective provirus frequencies following kidney transplant within therapy groups. Comparisons within therapy groups were further analyzed and subdivided into time bins. Each dot represents an individual within a time bin. Boxplots represent the interquartile range (IQR). The median is represented by the horizontal line in the box. The lower and upper whiskers represent 1·5 times the IQR beyond the quartiles. P values were estimated by Wilcoxon rank sum test.

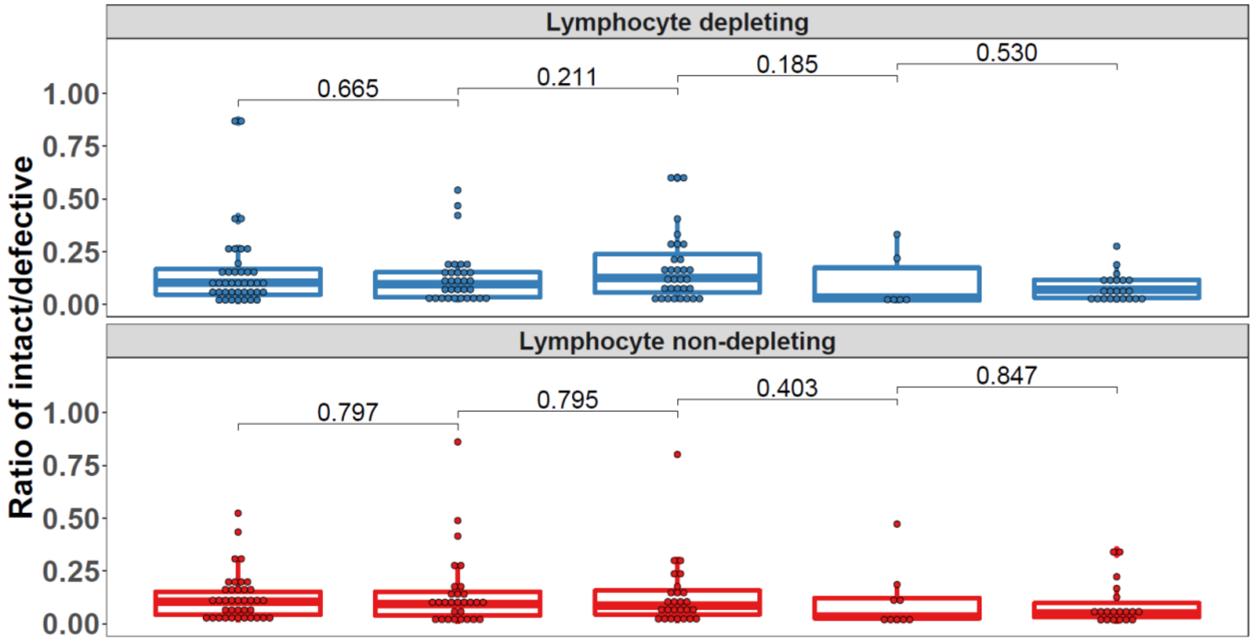

Supplemental Figure 7. Sensitivity analysis: Longitudinal trajectories of CD4+ T cells count per µL of blood following kidney transplant among patients received HIV- and HIV+ organs. (A) CD4+ T cell counts were measured longitudinally from times since transplant among patients who received **HIV- organs** and lymphocyte depleting or non-depleting treatment. Each line represents an individual, and each dot represents a time point. Blue and red lines represents the LOESS curve for lymphocyte depleting and non-depleting groups. Grey shaded areas represent the 95% confidence interval of the LOESS curves. (**B**) CD4+ T cell counts were measured longitudinally from times since transplant among patients who received **HIV+ organs** and lymphocyte depleting or non-depleting treatment. Each line represents an individual, and each dot represents a time point. Blue and red lines represents the LOESS curve for lymphocyte depleting and non-depleting groups. Grey shaded areas represent the 95% confidence interval of the LOESS curves.

A

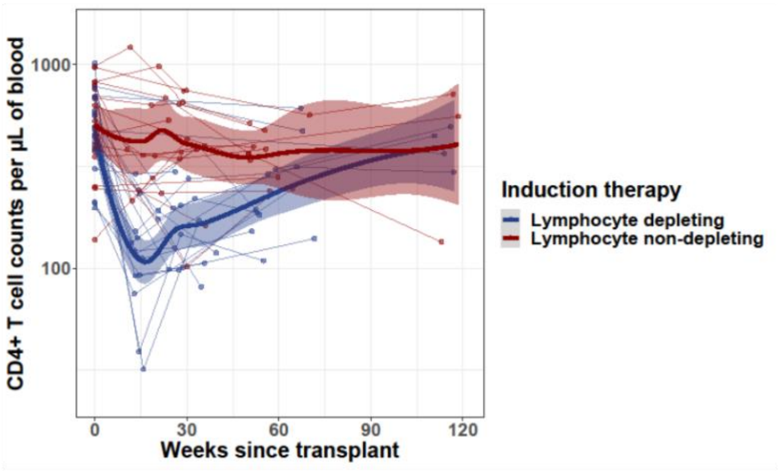

B

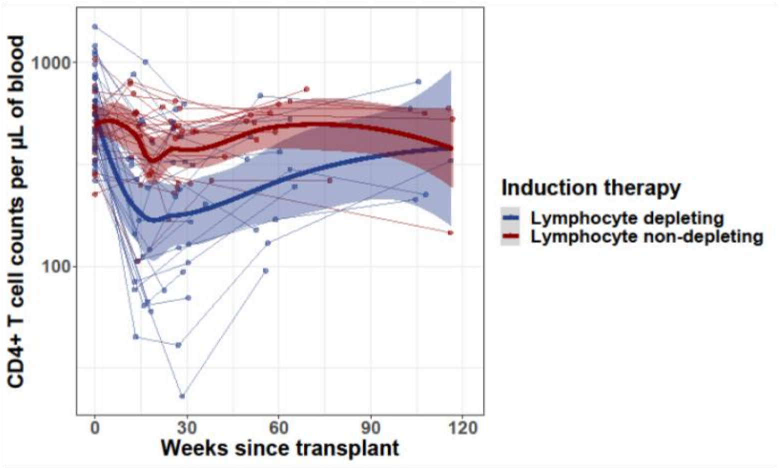

Supplemental Figure 8. Sensitivity analysis: Intact provirus frequencies per mL of blood following kidney transplant among patients received HIV- and HIV+ organs. (A) Intact proviruses were measured longitudinally from times since transplant among patients who received **HIV- organs** and lymphocyte depleting or non-depleting treatment. Each line represents an individual, and each dot represents a time point. Blue and red lines represents the LOESS curve for lymphocyte depleting and non-depleting groups. Grey shaded areas represent the 95% confidence interval of the LOESS curves. (**B**) Intact proviruses counts were measured longitudinally from times since transplant among patients who received **HIV+ organs** and lymphocyte depleting or non-depleting treatment. Each line represents an individual, and each dot represents a time point. Blue and red lines represents the LOESS curve for lymphocyte depleting and non-depleting groups. Grey shaded areas represent the 95% confidence interval of the LOESS curves.

A

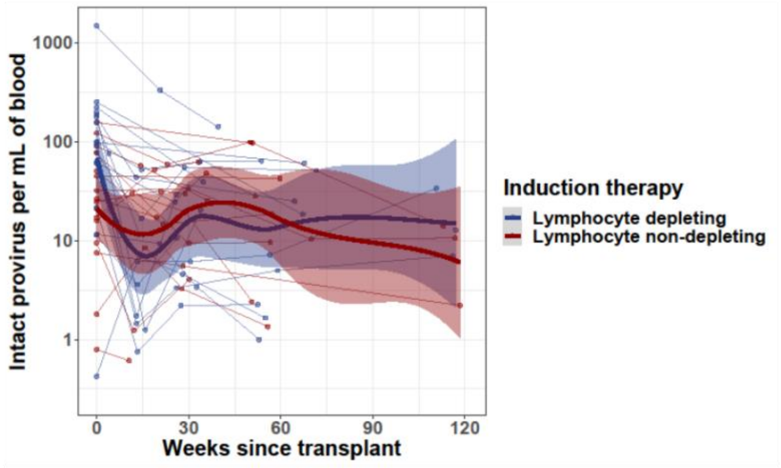

B

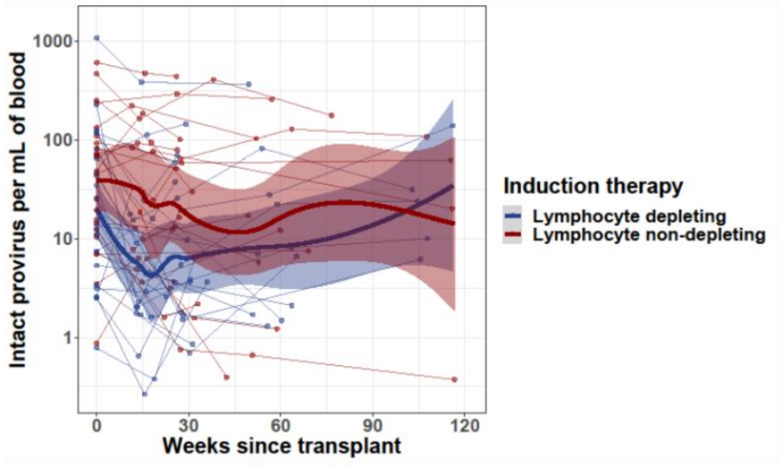

Supplemental Figure 9: TCRβ repertoire clonality, richness, turnover and newly detected and expanded clones following immunosuppression induction therapies. **(A)** Repertoire clonality, **(B)** repertoire richness, **(C)** repertoire turnover and **(D)** newly detected and expanded clones were measured since time since transplant among patients who received lymphocyte depleting compared to non-depleting therapies. Comparisons between therapy groups were further analyzed and subdivided into time bins. Each dot represents an individual within a time bin. Boxplots represent the interquartile range (IQR). The median is represented by the horizontal line in the box. The lower and upper whiskers represent 1·5 times the IQR beyond the quartiles. P values were estimated by Wilcoxon rank sum test. One patient was an outlier in (**D**) and therefore excluded from this analysis.

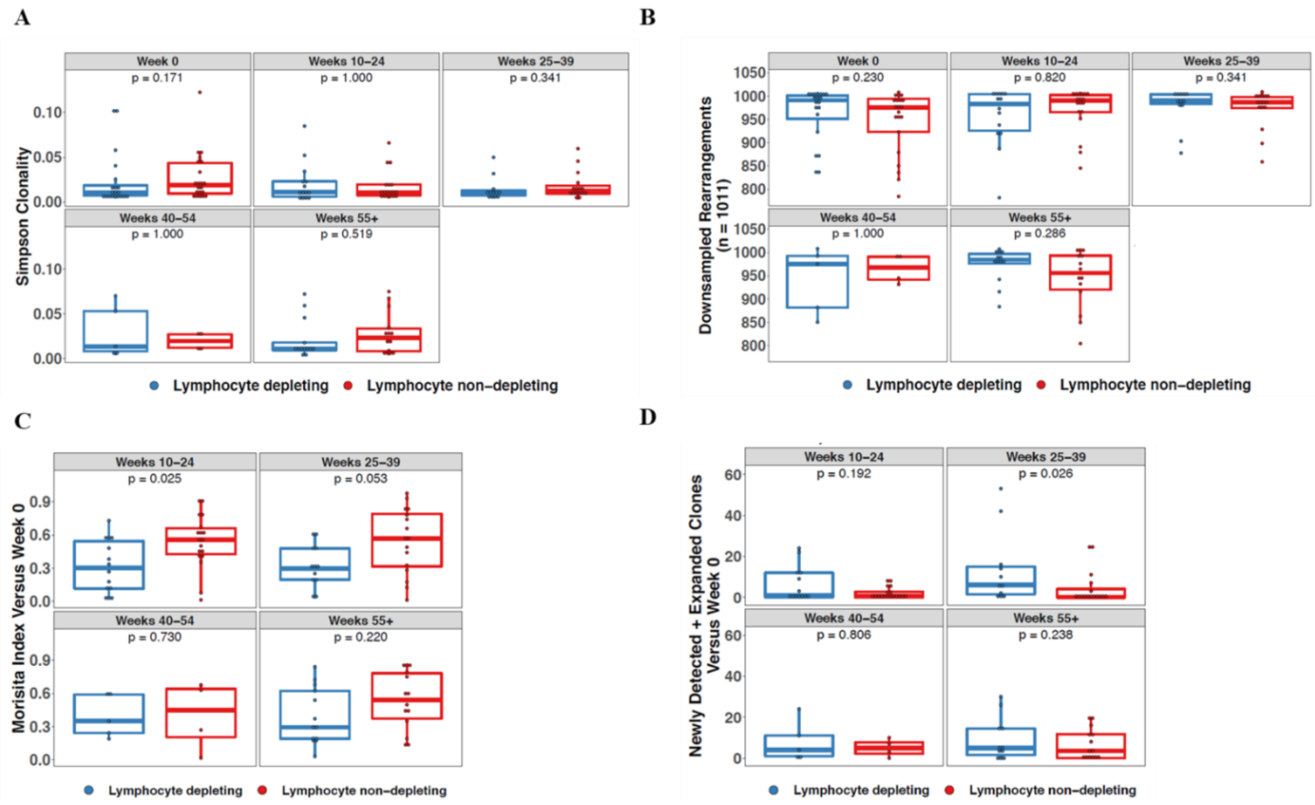

Supplement: Supplemental data [file jciinsight-7-162968-s092.pdf]
